# Cleveland neural engineering workshop 2017: strategic evaluation of neural engineering

**DOI:** 10.1186/s42234-019-0017-z

**Published:** 2019-01-30

**Authors:** Dustin J. Tyler, Christopher J. Czura, Jennifer French, Kip Ludwig, Kevin Otto, Forrest Pape, Cristin Welle

**Affiliations:** 1 Case Western Reserve University, 10900 Euclid Avenue, Wickenden 101, Cleveland, OH 44106-7207 USA; 2 Convergent Medical Technologies, Inc., New York, NY USA; 3 Neurotech Reports, San Francisco, CA USA; 4 University of Wisconsin, Madison, WI USA; 5 University of Florida, Gainseville, FL USA; 6 Medtronic, Minneapolis, MN USA; 7 University of Colorado, Boulder, CO USA

**Keywords:** Neural, Engineering, Strategy, Infrastructure, Advocacy, Rehabilitation, Nervous system

## Abstract

The Cleveland Neural Engineering Workshop (NEW) was established as a biennial meeting in 2011, with subsequent meetings taking place in 2013, 2015, and most recently, June 2017. This fourth biennial NEW was hosted by the Cleveland Advanced Platform for Technology National Veterans Affairs Center, the Functional Electrical Stimulation National Veterans Affairs Center, the Biomedical Engineering Department at Case Western Reserve University in Cleveland, Ohio, and Northwell Health’s Feinstein Institute for Medical Research of New York. The workshop connects leaders and stakeholders in the neural engineering community who are devoted to developing and deploying technological solutions to those with neurological disorders. The meeting in 2017 continued strategic conversations initiated at the third Cleveland NEW conference in 2015. The goal of the 2017 workshop was to was to determine specific actions by which the neural engineering community might advance the goals outlined in 2015, assess progress towards that plan, adjust as necessary, and establish continued strategic direction. This meeting report summarizes the outcomes.

## Introduction

“We envision a seamless integration between funding and regulatory agencies, and two-way communication between these organizations and the NE community, which speaks with one unified voice and is educated in regulatory processes. All stakeholders work together within an expedient, smooth regulatory and reimbursement ecosystem to bring the best neurotechnology products to patients.” Meeting Consensus Statement.

The first Neural Engineering Workshop was held in Cleveland, Ohio in 2011 (Cleveland NEW, 2018). Additional biennial meetings were held in 2013 (Cleveland New, 2013) and 2015 (Cleveland NEW, 2015). Participants in the NEW 2015 meeting developed a road map detailing strategic direction for stakeholders in the neural engineering community.

The fourth biennial Neural Engineering Workshop (NEW) was held June 2017 in Cleveland, Ohio. The Cleveland Advanced Platform for Technology National VA Center, the Functional Electrical Stimulation National VA Center, the Biomedical Engineering Department at Case Western Reserve University in Cleveland, Ohio, and Northwell Health’s Feinstein Institute for Medical Research of New York hosted the meeting. At this meeting participants reviewed the document produced in 2015 and continued strategic conversations. The goal of the 2017 workshop was to determine specific actions by which the neural engineering community might advance the goals outlined in 2015, assess progress towards the plan, adjust as necessary, and establish continued strategic direction. Specifically, the NEW 2017 conference sought to identify tangible short-term actions, “one-degree shifts,” that individuals might do to affect daily change towards identified goals.

**Fig. 1:**
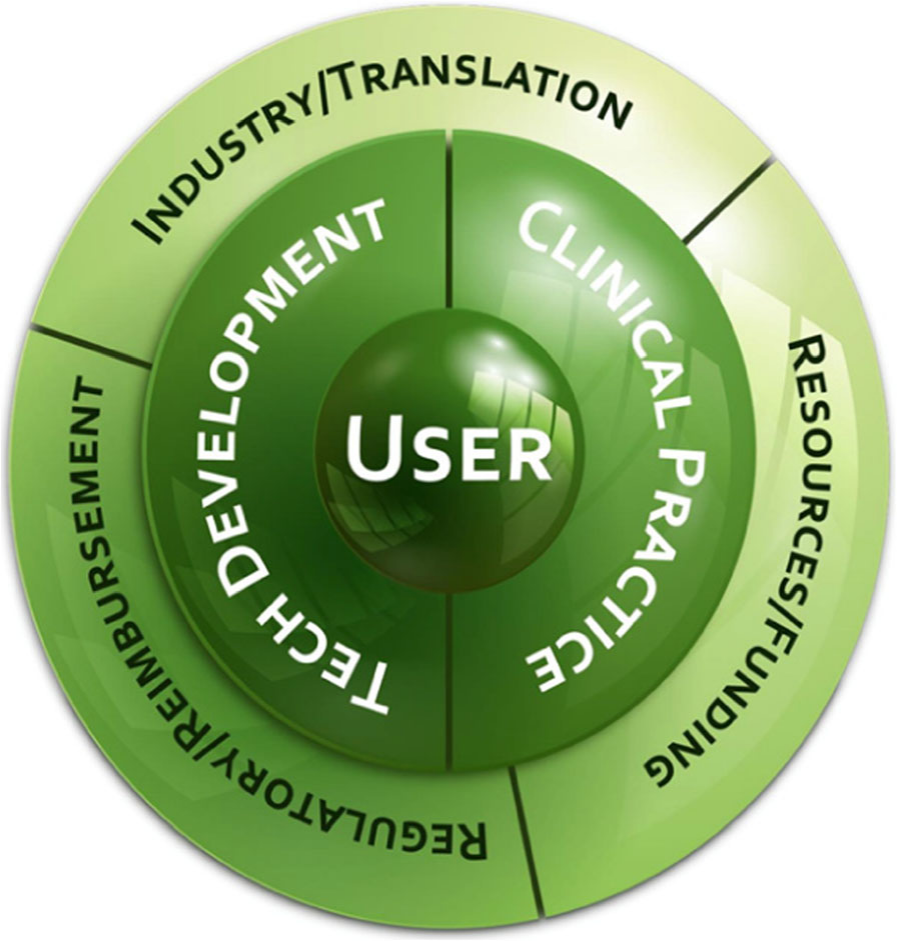
The relationship between the seven different themes identified in the Cleveland Neural Engineering Workshop meeting. The user/consumer plays a central role

Seven themes critical for success of the field were identified for the 2017 conference (Fig. 1). The themes are: Industry, Consumer, Funding, Reimbursement, Innovation, Clinical, and Regulatory. As indicated in Fig. 1, the user (or consumer) is a core element among the themes. The discussions, analyses, and specific actionable items from each theme are captured within this navigational document. This work serves as a grounding point for launching activities to advance strategic direction in each area. Each theme is described using the following format:

- **Current State**: Present considerations;
- **Key Factors:** Important elements in understanding challenges and discussions;
- **Vision:** Projected future development;
- **Goals:** Medium-to-long term action items to advance strategic direction; and
- **One-Degree Shifts:** Short-term/ongoing action items to advance strategic direction.

The output of the three-day workshop is provided in the table at the conclusion of this meeting report (Table 1). It is worth noting that much of the strategic direction within each theme centered on notions of education. A common thread of the attendees (Table 2) persisted throughout most of the discussions: neural engineering information needs to be shared and better understood among all stakeholders, including consumers, regulators, and innovators. It was the consensus that a more educated body of stakeholders would help drive innovation and more effective delivery of neural engineering technologies and treatments.

**Table 1:** Summary of vision and goals for each theme in the Cleveland Neural Engineering Workshop 2017 meeting

Vision	Goals
Theme: Industry	
We should make strides toward **fostering a community of trust and partnership** that accelerates academic, industry, and government collaboration to propel commercial translation of maturing scientific research and technology.	• Improve industry education and reduce silo effect among various groups and stakeholders • Encourage additional public-private partnerships (PPP) and/or expansion of these.
Theme: Consumer	
There must be opportunities to **engage the consumer more directly in the innovation process and to incorporate consumer data.** The consumer’s decision-making ability should be strengthened by equipping the consumer with more scientific information, therefore the field must develop ways in which interaction between innovators and assessors of the technology are informed by users of the technology.	• Write and submit open letters to the editors of a key technology/medical journal of needs statements regarding consumer/patient engagement within the neural engineering field. • Host working groups at other neural engineering related conferences.
Theme: Funding	
The high degree of collaboration necessary to gather funding and other resources should be present in the entire funding pipeline. We should push for **more collaborative and interdisciplinary science** at NIH and other organizations, including sources of “seed” funding to spur collaborations. Major resource providers such as venture capitalists should receive more education on the technology and its attendant use-cases and points of need, so that our work is understood as more than just a short-term investment or source of revenue. Similar bridges need to be built between pure science and business.	• Develop and share a comprehensive map of the funding ecosystem. • Across the discipline of neural engineering, begin publishing negative or contradictory results in bioRxiv as a resource for other researchers.
Theme: Reimbursement	
Recast the challenges of the reimbursement process in ways that better serve all stakeholders. Knowledge and understanding of CMS processes can guide us in the development of devices as early as the innovation phase. To develop a stronger and more communicative reimbursement process, we need to ask what we can do for CMS and how CMS can serve us better. We must help CMS better understand the social value of our devices.	• Make our community aware of CMS. • Build a relationship with CMS. • Fund a fellow at CMS that serves as a bridge between our communities.
Theme: Innovation	
**Increase patient agency** by developing systems that are sustainable, secure, closed-loop, minimally or non-invasive, and responsive. Neuroengineers can **optimize risk and reward** by expanding knowledge of the physiological basis for neural disorders and developing a nuanced classification strategy for patient selection. Neuroengineering can both show us and guide us toward the future, and we must innovate cultures of ethics and inclusion within the field and among those who regulate or benefit from its technologies.	• Create a global neural engineering forum. • Increase communications about neural engineering innovations. • Increase diversity as a means for innovation.
Theme: Clinical	
We envision clinical efficiency in developing and deploying breakthrough solutions that maximize self-agency and balance risk with reward to improve the quality of life for individuals living with diseases or disorders of the nervous system. The goal is to help restore users’ self-agency and participation in their communities of choice through a collaborative, inclusive, multidisciplinary, biobehavioral approach.	• Improve bi-directional interactions between neural engineers and clinicians. • Define views on “augmentation;” a neuroethical framework.
Theme: Regulatory	
We envision a seamless integration between funding and regulatory agencies, and two-way communication between these organizations and the NE community, which speaks with one unified voice and is educated in regulatory processes. All stakeholders work together within an expedient, smooth regulatory and reimbursement ecosystem to bring the best neurotechnology products to patients.	• Develop an IDE template or examples to share within the community. • Recommend and identify inter-agency liaisons between federal funding agencies and the FDA. • Write consensus responses to FDA guidances related to the neural engineering community. • Write a formal request that NIH support regulatory science requests for application (RFAs).

**Table 2:** Attendees of the 2017 Cleveland Neural Engineering Workshop meeting

Attendee	Company	Attendee	Company
Ajiboye, A. Bolu	Case Western Res Univ	Moritz, Chet	University of Washington
Ashmont, Kari	NIH/NINDS	Moynahan, Megan	Inst for Functional Restoration
Bagen, Susan	Micro Systems Technologies	Muni, Robert	ImpactMed
Bardot, Dawn	Med Device Innov Consort	Otto, Kevin	University of Florida
Baum, Robin	Case Western Res Univ	Pancrazio, Joseph	UT Dallas
Biederman, Lucy	Case Western Res Univ	Pannu, Satinderpall	Nevro Corporation
Bourbeau, Dennis	Cleveland FES Center	Pape, Forrest	Medtronic
Calton, Robert	Case Western Res Univ	Peckham, P. Hunter	Metrohealth Rehab Inst
Cartellone, Mark	SmartShape Design	Peterson, Erik	Medtronic
Charkhkar, Hamid	APT Center	Platt, Jo Jo	Center for Bioelectr Med
Christie, Breanne	Case Western Res Univ	Puerta, Margot	Feinstein Inst for Med Res
Cornwell, Andrew	Cleveland FES Center	Ramdeo, Richard	Feinstein Inst for Med Res
Cuberovic, Ivana	Case Western Res Univ	Richardson, Mark	University of Pitt School of Med
Cunningham, David	Cleveland FES Center	Rowan, Robert	Case Western Res Univ
Czura, Christopher	Feinstein Inst for Med Res	Schearer, Eric	Cleveland State University
Datta, Proyag	Second Sight Medical Products	Schiefer, Matthew	APT Center
Dorval, Chuck	University of Utah	Shire, Doug	APT Center
Durand, Dominique	Cleveland FES Center	Shoffstall, Andrew	APT Center
Ereifej, Evon	APT Center	Sohal, Harbaljit	Feinstein Inst for Med Res
French, Jen	Neurotech Rprts/Neurotech Net	Straka, Malgorzata	Northwell Health
Ganzer, Patrick	Battelle Memorial Institute	Sweet, Jennifer	University Hospitals
Graczyk, Emily	Case Western Res Univ	Tan, Winny	Imec
Hermann, John	Case Western Res Univ	Timco, Paula	nuboHEALTH, LLC
Hess-Dunning, Allison	APT Center	Triolo, Ron	APT Center
Kirsch, Bob	Cleveland FES Center	Tyler, Dustin	Case Western Res Univ
Kozai, Takashi	University of Pittsburgh	Vasudevan, Srikanth	U.S. FDA
Kusiak, Audrey	Department of Veterans Affairs	Wagenaar, Joost	Blackfynn Inc.
Langhals, Nicholas	NIH/NINDS	Wang, Jon	ImpactMed
Levy, Todd	Northwell Health	Weisberg, Greg	SmartShape Design
Ludwig, Kip	Mayo Clinic	Welle, Cristin	Univy of Colorado
Lujan, Luis	Mayo Clinic	Wheeler, Tracey	Craig H. Neilsen Foundation
Makowski, Nathan	Metrohealth Medical Center	Widge, Alik	Massachusetts General Hospital
Marasco, Paul	Cleveland Clinic	Williams, Matt	Cleveland FES Center
Mazanec, Paul	Envoy Medical	Xue, Shannon	ImpactMed
McIntyre, Cameron	Case Western Res Univ	Zariffa, Jose	Univ of Toronto
Michaels, Bob	Case Western Res Univ	Zbrzeski, Adeline	Synapse Biomedical
Mohseni, Pedram	Case Western Res Univ	Zhang, Mingming	Battelle Memorial Institute

## Theme: Industry

### Current state

The term “industry” is shorthand for a complex ecosystem of agencies, companies, innovators, investors, regulations, and customers. Navigating the industry can be challenging, even for the most experienced people. The translation process, moving an innovation through each of the stages between the workbench and the patient’s bedside, is time and resource-intensive. Companies may also struggle to meet insurers’ requirements and metrics for reimbursement. This can pose a substantial barrier to entry for smaller or less well-funded companies, often independent of their products’ viability or potential market share.

Large companies often have the institutional knowledge to guide their innovations along but may lack the agility and creativity of smaller start-ups. New companies may struggle to stay funded while scaling the steep learning curve inherent to the industry ecosystem. Academics and clinicians are in particular need—and lack access to—solid, consistent, and current navigational training. Emerging scholars and practitioners in graduate programs also need introductory education on the existence and uses of quality systems in industry.

When programmatic successes happen, as with the Brain Research through Advancing Innovative Neurotechnologies (BRAIN) public-private partnership (Author, 2016), the initiating agencies deserve to receive feedback illustrating what’s working well and outlining opportunities for further expansion or improvements. Productive collaborations can be encouraged through open and multi-directional communication, or they can be stymied by legal and procedural impediments to knowledge sharing among colleagues or peers across the industry.

### Key factors

#### Improving collaboration

Intellectual property rights and funding rules can distort or inhibit the flow of information among people and within the larger ecosystem. As a result, individuals feel unable or uncomfortable communicating openly and honestly with one another. This may be due to siloed activities or isolation from other areas of the ecosystem. It may also be because people logically suited for collaboration with one another are trying to achieve multiple and sometimes conflicting financial, competitive, legal, or other outcomes simultaneously.

A focus on sharing granular information between industry partners, agencies, users, and researchers should be preferred to larger, systemic approaches to communication. We need to strike a balance that turns partnerships and community into a competitive advantage over isolation and secrecy.

#### Navigating complex terrain

The work of improving communication and educating innovators on effective navigation through the industry ecosystem should begin early in one’s career (in graduate programs and other similar sites). Education and effective communication are processes rather than goals. The process must be encouraged for each innovator as they move into the field and shift from mentee to mentor. This will help ensure a robust and self-sustaining meta-process of communication and institutional knowledge transfer throughout the ecosystem. Enhanced education among innovators relating to navigation of these complex structures could also yield improved outcomes at each waypoint within the system.

#### Expanding and enhancing public-private partnerships

Hand-offs from the laboratory to commercialization involve large shifts in technology, tools, and clinical goals with each new link in the chain. The timeframe for clinical studies is measured in years and decades. The necessary funding is high and commercialization processes are highly inefficient with significant potential for failure.

### Vision

We should make strides toward fostering a community of trust and partnership that accelerates academic, industry, and government collaboration to propel commercial translation of maturing scientific research and technology.

### Goals (2–5 years)

#### Improve industry education and reduce silo effect among various groups and stakeholders

- Design and run NANS/NIC workshops to give vision on how to improve research and clinical translation.
- Design and run crash courses for entrepreneurship.
- Develop fellowships or other mechanisms that train younger generations of researchers to make sure they gain exposure to clinical work, industry and regulatory needs, and other expectations of their future careers beyond scientific and technical matters.
- Create and promote joint PhD/MS co-ops & internships.
- Engage with members of Congress (such as by inviting them to conferences) to educate them on the benefits of the research and development being done in neural engineering.

#### Encourage additional public-private partnerships (PPP) and/or expansion of these

- Encourage outreach to clinicians directly. Clinicians tend to work with industry more regularly than academics and can bring knowledge and insights from those interactions.
- Academics should be encouraged to explore public-private partnerships more, as many are simply unaware of these opportunities at the NIH.
- Share success stories. To educate industry partners, find stories that illustrate how far one can go with federal funding.

### One degree shifts

#### Education

Education is the chief concern in the Industry and Translation workgroup.

- Improve awareness of available navigation-oriented training by investigating C3i and I-CORP programs and compiling the details on a common portal.
- As a precursor to potential development of a commonly used program or set of resources, survey Biomechanical Engineering department chairs about courses, materials, and other educational tools they use to teach their students about quality systems and other practical aspects of working in or with industry.
- Expand awareness of public-private partnerships and amplify what’s working by seeding an upcoming NANS panel with PPP successes and opportunities.

#### White paper

Draft a white paper urging the NIH to expand its BRAIN public-private partnership (or to create others) and enumerating the positive outcomes of the program so far. Ideally, this white paper would be signed by as many Cleveland NEW participants as possible.

## Theme: Consumer

### Current state

The “consumer” refers not only to the patient, but also the caretaker, support network, and advocate community centered around the “end user” of engineered neurotechnology devices. The indications for neural interface technology are expanding rapidly, ranging across conditions such as chronic pain, sleep apnea, movement disorder, amputation, spinal cord injury, and a rapidly growing list of conditions. The consumer requirements for each of these areas may vary widely. Consumers with indications like sleep apnea and chronic pain are generally healthy and lead active lives. They would prefer technology that is ‘invisible,’ not interfering with everyday activities or requiring any user attention. Other consumers with indications such as spinal cord injury or stroke have a much greater functional impairment and will develop a more extended relationship with their neural technology. The collective need and experience of the consumers should inform the decisions and direction of neural technology development.

Neural engineers tend to have a unique and potentially fruitful relationship with users of devices. The users tend to be extraordinarily engaged in the use of their devices, with a direct understanding of the need for the therapy the device provides. Additionally, consumers of these devices tend to care about the use and success of their devices and to use them in real-world, daily life operations. The strength of their engagement and the strong feedback that consumers have the potential to provide means we need to make greater use of consumer feedback at every stage of the process, from innovation to clinical trial design to the regulatory approval process.

Because of the special relationships users have to neural devices, an emerging trend in neurotechnology is the movement from patient information being a collection of anecdotes that are not sufficiently data-driven, to patient information being solicited, collected, and used as scientific data for decision-making purposes at every stage of development of the neural device. Developing stronger pathways for patient input, including patient preference information (PPI) and patient-reported outcomes (PRO), serves all stakeholders. Those pathways serve neural engineers by reducing the cost of evidence gathering and providing more robust evidence throughout the development of devices. Stronger pathways for the incorporation of PPI and PRO serve patients because they result in devices that better meet their needs.

A related trend is the incorporation of Patient-Centered Outcomes Research (PCOR) in the development and implementation phases of neurotechnology devices, which involves asking questions about patients at scale. PCOR considers issues including patient survival and quality of life, barriers to implementation, and availability of the device across various populations, among other factors.

As in NEW 2013 and 2015, it was noted that consumers of neurotechnologies tend to be self-educated regarding devices, relying on the Internet and anecdotal information. Those types of information should be supplemented with scientific data to increase users’ engagement with and adoption of these technologies. A consensus opinion emerged from the discussions:

The more educated and engaged consumers of our devices become, the better positioned they will be to advocate on behalf for themselves, improvements to neural engineering technologies, and the broader neural engineering community.

### Key factors

#### Identity of the “consumer”

There is a debate surrounding which word to use when referring to the consumer. This stems from the various roles individuals play in the development of neural engineering technology - from provider of key feedback regarding the device to patient advocate. Some members of the workshop group suggested the term “consumer” may have a negative connotation for clinicians, because it sounds as if the person being treated is consistently being sold something across the course of their treatment.

Clinicians sometimes dislike the term “stakeholder” for the same reason, associating that term with capital-related rather than health-related aims. Therefore, “user” can be preferable, as it targets the person under treatment’s experience with the device, which is of primary interest in this context to the engineer of the device, the user of the device, and the clinician. Some clinicians may prefer the term patient. However, members of the workshop group pointed out this term can be less accurate than others, because users are not always under care of clinicians, but they are always consumers, at least potentially.

Group members concluded that in general the terms “user” and “consumer” were preferable when referring to those who use or need neural engineering devices, with both terms being used deliberately and specifically. The term “user” is preferable in situations that directly refer to the consumer’s experience with the device, while the term “consumer” is more generally applicable.

### Vision

There must be opportunities to engage the consumer more directly in the innovation process and to incorporate consumer data. The consumer’s decision-making ability should be strengthened by equipping the consumer with more scientific information, therefore the field must develop ways in which interaction between innovators and assessors of the technology are informed by users of the technology.

### Goals (2 to 5 years)

#### Write and submit open letters to the editors of a key technology/medical journal of needs statements regarding consumer/patient engagement within the neural engineering field

- Assess journals in the field to determine which would be the most appropriate target for letter(s). There may be different letters based on the differences in the populations of the underlying medical indications.
- Outline and draft the letter(s).
- Provide collective and consistent communication regarding the letter(s).
- Widely communicate the letter’s message.

#### Host a working group at other neural engineering related conference

- Address consumer/patient engagement needs statements to share with targeted funding agencies.

## Theme: Funding

### Current state

The central concern is acquiring funding for promising technologies, which can then be more thoroughly researched, developed, and understood. Securing and allocating resources is not just a matter of math; it is an act of rhetorical persuasion, which helps the medical and engineering communities to both explain and understand what types of technologies are important or worth future investment of time, money, and other resources.

Long-term financial support systems are inconsistent around the world, and therefore collaboration remains a key component among investigators, funders, and industry. The larger goal, supported but not fully addressable by this forum, is the development of a comprehensive funding landscape that cultivates research along the continuum, from initial discovery to consumer-focused translation and onward to sustainable commercial dissemination.

### Key factors

#### Document the resource ecosystem

Many researchers are unaware of the full suite of resources they could access, due in part to the fragmented and disparate nature of these resources. A regularly updated map or catalog of funding opportunities, particularly those that are under-utilized, could be of significant benefit. A matchmaking resource, which connects investigators with others doing similar work, or with sponsors and investors, could also prove useful. Training on the commercialization process is inconsistent among new and emerging investigators. A catalog of available training materials or a recurring session at conferences could save many researchers the burden of reinventing the wheel. For any or all of these meta-resources, wikis or other open-source document management tools could be a low-cost way to distribute the information gathering workload.

#### Increase efficiency of research projects

One way to make funding dollars stretch further is to maximize clinical trial outputs, including (but not limited to) accessing data from failed trials or unsuccessful portions of larger research projects. Some portion of clinical trials will fail. Failed trials, however, can still provide valuable information by understanding *why* they failed. For example, if there are key flaws in the animal model used in pre-clinical trials, this information can improve the pre-clinical model and/or prevent future clinical attempts based on flawed supporting data. Information about the failure could help investigators avoid costly delays, mistakes, or unproductive avenues of study. One possibility is to ask researchers to include more details about the unsuccessful portions of trials in addition to writing up their successes, possibly as an appendix or supplement to the main article. We would likely need to ask journal editorial boards for buy-in on this change in practice so reviewers didn’t flag the new material as unnecessary.

Another possibility is to follow the arXiv model used by the physics community, in which unpublished articles and related data are stored in a commonly accessible online framework (https://arxiv.org). The biological sciences equivalent, which went online in 2013, is called bioRxiv (https://www.biorxiv.org).

### Vision

The high degree of collaboration necessary to gather funding and other resources should be present in the entire funding pipeline. We should push for more collaborative and interdisciplinary science at NIH and other organizations, including sources of seed funding to spur collaborations. Major resource providers such as venture capitalists should receive more education on the technology and its attendant use-cases and points of need, so that our work is understood as more than just a short-term investment or source of revenue. Similar bridges need to be built between pure science and business.

### Goals

#### Develop and share a comprehensive map of the funding ecosystem

- Communicate funding match-up information among sponsors, investors, and researchers in neural engineering.
- Make use of existing social media platforms for maximum efficiency in sharing information, rather than attempting to create a new platform or distribution method.

#### Across the discipline of neural engineering, begin publishing negative or contradictory results in bioRxiv as a resource for other researchers

- Based on research conducted by the group in the Thursday afternoon session, it is believed that publishing in bioRxiv will not prevent later publication in typical peer-reviewed journals. However, further research and confirmation is desired.
- Spreading the word at conferences and through other community-based media will help to encourage this new practice as a standard.
- The NIH requires making all data collected from a completed grant-funded study to be readily available to the public within one year; funding agencies like NIH could be encouraged to mandate the use of bioRxiv as a repository for the full dataset.

### One degree shifts

Education and making better, more efficient use of available resources are the dominant themes of the deliverables that emerged from the Funding workshop sessions.

- Conduct an inventory of existing funding opportunities.
- Create a LinkedIn group named “Neural Engineering Community” and invite members to join.
- Collate data on funding sources (including date, range of amount, state or federal source type, and other appropriate attributes) and post the resulting documents to the LinkedIn group.
- Compile a monthly or quarterly recap of funding opportunities, along with a narrative explanation of how those opportunities can or should be accessed; post the compilation to LinkedIn.
- Conduct additional research to ensure that relevant publishers are okay with pre-publishing in bioRxiv.
- Create or request a custom category or channel called “Neural Engineering” in bioRxiv.
- Request to publish negative, contradictory, or other under-reported results to bioRxiv.

## Theme: Reimbursement

### Current state

In 2017 for the first time at Cleveland NEW, reimbursement was a standalone theme, separate from the regulatory theme. In 2013 and 2015, discussion in the reimbursement/regulatory group centered on issues of regulation, focusing on increasing communication with and knowledge of the operations of the Food and Drug Administration (FDA).

The increased discussion, concern, and interest among Cleveland NEW workshop participants from 2013 to 2017 regarding reimbursement, and the need to create a separate theme for reimbursement, attest to the volatility in this area and to its significance to neural engineering technology.

The importance of educating ourselves and our community about the Centers from Medicare and Medicaid Services (CMS) reimbursement process can hardly be overstated, because CMS approval is often the determining factor in whether a device reaches commercialization. Innovations that have received approval from other bodies, like the FDA, but are then stymied at the CMS level, virtually guaranteeing that they will not reach commercialization. If translation is the goal of a research effort, understanding the CMS process will guide efforts towards solutions that may reach patients rather than fail at market entry after 10’s to 100’s of millions spent on research.

As one workshop participant noted, the importance of CMS approval to the success of a new neurotechnology means that it would be appropriate to begin to provide prospective neural engineers with information about the CMS process during undergraduate engineering classes. In the 2015 reimbursement/regulatory workshop, participants noted as major challenges a lack of communication with CMS and a lack of knowledge regarding the CMS decision-making process. These conditions still hold in 2017. Participants voiced confusion regarding the written CMS guidelines, which are difficult to understand and follow. Additionally, alongside the rapid changes in technology noted in 2015, possible policy changes in the current environment present uncertainty.

The Affordable Care Act included reimbursement policy changes that impacted CMS and the insurance industry, which follows the example of CMS in determining whether to assign reimbursement codes (Author, 2010). As neural engineers who seek reimbursement codes for new technologies continue to acclimate to these changes, there looms the possibility of an entirely new set of policy changes, should the Affordable Care Act be repealed under the current administration.

### Key factors

Participants noted similarities between the current state of communications with CMS and the regulatory environment about the FDA ten years ago. The improved FDA regulation process could provide a blueprint for increased and improved interaction with CMS. Among the lessons learned from the FDA example is our efficacy as a group. When we work and speak on behalf of the field, rather than on behalf of an individual company or technology, our voice is stronger, more unified, and more convincing.

The lack of timeline regarding the reimbursement process can be detrimental to innovation. Participants noted that they were unsure at what point in the process CMS should be brought in. At various points in the discussion, participants asked when, how, and why do we engage CMS, who do we talk to, and how often during the process?

The breadth of those questions suggests both our need and desire for more open lines of communication with CMS. This lack of knowledge likely moves in both directions. To receive the clarity and information we need from CMS, we also need to provide them with clarity and information about our work and its importance.

In addition to communication-related challenges, other challenges presented by the current reimbursement environment include the increasingly high costs associated with generating evidence and the increasing demand for evidence to receive reimbursement approval.

### Vision

Recast the challenges of the reimbursement process in ways that better serve all stakeholders. Knowledge and understanding of CMS processes can guide us in the development of devices as early as the innovation phase. To develop a stronger and more communicative reimbursement process, we need to ask what we can do for CMS and how CMS can serve us better. We must help CMS better understand the social value of our devices.

The reimbursement group addressed underlying questions about how innovative technology can be moved through the CMS process in ways that better serve the technology and the patient. For example, one participant questioned what role the patient plays in driving CMS decisions. PRO and PRI could be better used to in the process, reducing evidence costs, improving outcomes, and increasing the chance of CMS approval.

Another source of cost effective, patient-centered data is the U.S. Veterans Affairs office, given its unique system of health records and clinical trials.

### Goals (2 to 5 years)

#### Make our community aware of CMS

We need to increase awareness and understanding of the reimbursement process. Although most participants noted that it has been difficult to communicate with CMS, others noted that CMS does in some ways reach out to innovators and attempt to provide information and education, including holding a public forum once a month and featuring webinars and tips on its website.

The first task in educating our community is to compile and share the resources currently available regarding reimbursement, such as those on the CMS website. The second task in educating our community is to publish a paper containing a variety of instances of illustrative neural engineering devices that successfully received CMS coding and those that did not. There was discussion regarding how best to disseminate this information, with the conclusion that the CMS-related resources should be posted to ClevelandNEW.org and LinkedIn and that the case studies paper should be published in an open access journal, with the goal of reaching as many people in the community as possible.

### Build a relationship with CMS

Building a relationship between the neural engineering community and CMS means advocating for ourselves as well as connecting with organizations and individuals who can advocate on behalf of the neural engineering community to CMS.

Relevant organizations might include clinical medical societies like the American Medical Association and patient advocacy groups; relevant individuals might include high-level clinical trial experts and congressional representatives who have significant neuroengineering activity in their districts and/or sit on committees related to healthcare regulation and reimbursement. We need to invite these and other interested parties to understand our challenge and work together with us to solve it. When reaching out to individuals at CMS, we recognize the distinction between the national-level organization and local- and regional-level hospitals and insurance companies with which CMS contracts.

Our efforts will begin at the local and regional levels because this offers an increased ability to connect with individuals already within our networks with an awareness or understanding of the neural engineering community.

#### Fund a fellow at CMS that serves as a bridge between our communities

“You never really know a man until you understand things from his point of view, until you climb into his skin and walk around in it.” (Lee, 1960)

To understand CMS and its impact on our community, we should consider methods of collaborative interaction. For example, there would be value in embedding a member of the neural engineering community within the CMS community. One such example could be a neuromodulation fellow that is established to work at CMS and serve as a bridge to be mutually beneficial to both communities.

## Theme: Innovation

### Current state

There is a broad and accelerating interest from funders, including the BRAIN Initiative, Innovation Challenge, as well as industry, to produce innovative NE technologies. Established entities like Medtronic, Boston Scientific, and St. Jude/Abbot are starting to see competition in the form of emerging groups such as Neuralink, Kernel, even Facebook and Google.

Innovation is moving towards having neuromodulation transition toward minimally or non-invasive technologies, including recent advancements in non-invasive DBS, transcutaneous spinal stimulation, modulated and focused ultrasound, and optical/magnetic stimulation. There is also a shift towards understanding and developing multi-modal neural devices, which collaborate with biological approaches such as electrical optical activation of stem cells.

Progress since 2015 has started to “close the loop” between recording and stimulation, with advances in brain-controlled muscle, spinal, and deep brain stimulation. Emerging progress in 2017 includes bi-directional interfaces (human trials), BCI decoding and sensory feedback from limbs, as well as prosthetic limb interfaces with motor decode and sensory feedback.

### Key factors

#### Identify several grand challenges and their barriers

As the field continues to innovate, we must consider the challenges facing innovation. By defining the obstacles, such as intellectual property issues that discourage open and innovative dialog, we might be better able to meet them head-on and find appropriate solutions to minimize or mitigate them.

#### Create common platforms that enable innovation

As collaboration and education are central components to innovation, creating a platform in which researchers, clinicians, engineers, and other stakeholders can communicate would drastically open the pathways to innovate technologies that are represented through these various groups’ best practices. It is critical, therefore, that we promote the development and use of such a common platform.

#### Create a culture of innovation that mitigates risk and allows us to learn from failures

Aligned with the themes of education and collaboration, an overall movement towards innovating together would allow us to better deal with risk and failure.

### Vision

Increase patient agency by developing systems that are sustainable, secure, closed-loop, minimally or non-invasive, and responsive. Neuroengineers can optimize risk and reward by expanding knowledge of the physiological basis for neural disorders and developing a nuanced classification strategy for patient selection. Neuroengineering can both show us and guide us toward the future, and we must innovate cultures of ethics and inclusion within the field and among those who regulate or benefit from its technologies.

### Goals (2 to 5 years)

#### Create a global neural engineering forum

Connect neural engineering researchers and clinicians to encourage sharing of data and best practices.

- Develop a searchable laboratory website template that could be linked to a common interface for identifying experts in the domain of neural engineering and sharing best practice between them.
- Garner interest in community-generated documents for sharing knowledge that facilitate discovery and translation (e.g. library of FDA-approved materials for implanted devices).

#### Increase communication about neural engineering innovations

- Consider collaboration on a JNE review paper for education of students or industry.
- Create “conversation articles” in field publications.
- Promote collaboration at other workshops and meetings (NANS/NIC).

#### Increase diversity as a means for innovation

Communicate excitement in the field to engage new collaborators and communities.

### One degree shifts

#### Create a JNE paper on ‘The therapeutic potential of neurotechnology’.

- Convey emerging potential of neurotechnology to improve health and neuroscience discovery.
- Call to action for clinicians and researchers to engage with diverse fields and push neurotechnology forward for social good.
- Inspire next generation of neural engineers to join field and drive innovation.

## Theme: Clinical

### Current state

Neural engineering (NE) science has reached new frontiers. It has the potential to help ameliorate chronic diseases such as Tourette’s Syndrome and Parkinson’s Disease, and to be combined with other modalities (such as artificial intelligence) to manage conditions such as obesity, diabetes and heart disease.

In this new research landscape, neuroengineers, clinicians and patients work together to develop neural devices that achieve optimal benefits for patients and caregivers. Patients are no longer merely the hosts or subjects – they are becoming co-scientists in the innovation process. There is an increasing need for clinicians to receive patient feedback and data, and this workshop has identified several key reasons why we need more input from patients. Since clinicians and researchers don’t always know what patients need, it also allows clinical assumptions to be tested and adjusted based on real-world use or patient preference.

Treating neuroscience research patients as co-scientists in their own protocols can have beneficial psychological effects, can result in better therapy compliance, and could contribute to a successful outcome for the patient and the project. However, the NE community must balance the need for patient input with the host of global, cultural and gender-related differences that will affect preferences and feedback.

Financial constraints are an obstacle for the NE clinical community because many of their innovations are highly individualized and not designed to be immediately scalable for commercialization.

The 2017 clinical workgroups reinforced the belief stated in 2015 that in this new era of innovation, physicians and neuroengineers are partners on the “clinical team.” This theme was repeated throughout the sessions. Researchers can feel distanced from the clinical aspects of the protocols, especially what’s working well and what isn’t, and the clinicians feel that a deeper understanding of the engineering aspects will inform their clinical procedures.

To improve understanding of every team member’s capabilities, neural engineers should engage with the medical field they’re trying to impact, and clinicians should spend time with neuroengineers. In addition, both need to spend time with end-users, caregivers, and physical and occupational therapists to better understand the synergies, mechanics, and therapeutic aspects of device/patient interactions. “Opening up” to other collaborators teaches all team members about how they fit, what others contribute, and how important each contribution is.

There is one clinical environment in which neuroengineering challenges are mitigated. The Veteran’s Administration (VA) is a unique key player in the NE community. Its research and services are fully dedicated to injured veterans who have served honorably in the United States military. “The VA takes care of its people – period,” one workgroup member stated. As part of its mandate, the VA must maintain specialized treatment and rehabilitation programs for spinal injuries, blindness, amputations, mental illness, and other serious service-connected health conditions. As a result, many typical barriers to research and treatment, including tight funding limitations, are not present, and VA neuroengineers and clinicians enjoy greater flexibility and support to develop and deploy new technologies. Another benefit of the VA system (and not typical in the public sector) are the variety of ancillary services, training and support provided to caregivers, whom they value as critical ongoing support for their veterans.

Fortunately, the VA also cares about translating successful technologies out to non-VA patients. This makes them a valuable partner and ally for the NE community. The VA is also an anomaly with respect to development costs and budgets, since cost is less important to them than providing optimal patient care. VA researchers also have protected research time – they can investigate a variety of solutions without as much concern for marketability or commercial value.

### Key factors

#### Value-based care

What are the most cost-effective technologies? Cost only goes down as demand increases, and the NE field is designing customized devices for small, specific populations, so their commercialization value is questionable.

#### American distrust of science and scientists

This increasing negative perception is affecting the NE research community. Workgroup members discussed the need to educate the different social, generational, political and ethnic communities and rebuild trust by sharing good information, answering questions and maintaining an open line of communication.

#### Increasing global perspective

Healthcare, both economy and practice, is adopting a more global perspective. Healthcare is not a single country’s challenge. The approach to healthcare and delivery of technological solutions, however differs widely across the globe. Continued dialog and solutions require increased focus on global context.

#### Data sharing

The enabling of widespread data sharing could greatly impact the success and efficiency of trials. The total NE patient population is relatively minuscule, so an enlarged data pool would provide broader and richer study information for better decision-making. This could help investigators focus on the most productive research, avoid duplicating other work, and speed successful product development.

#### Multi-modal support

An increase of promotion of this style of support: including family, friends, counselors, clinicians, occupational and physical therapists, and even healthcare aides, will help to achieve lasting self-agency and success. Unfortunately, long-term support systems are inconsistent in different geographies and healthcare networks. When a grant runs out, what happens to the patient? Some patients are great at adapting; others are not.

#### A neuroethical framework

Despite many questions circulating about neuroethics and its place in the work of the NE community, no framework has yet been established to navigate the murky ethical issues. Where should the line be drawn between ‘rehabilitation’ and ‘augmentation’? How do we categorize memory implants for Alzheimer’s patients, and devices such as exoskeletons? Should functionality be delivered at any cost? How should the community support hundreds of people with fully implanted devices after the trials are over? These issues and others will continue to emerge, and they require consensus guidance upon which to base ethical decisions.

### Vision

We envision clinical efficiency in developing and deploying breakthrough solutions that maximize self-agency and balance risk with reward to improve the quality of life for individuals living with diseases or disorders of the nervous system. The goal is to help restore users’ self-agency and participation in their communities of choice through a collaborative, inclusive, multidisciplinary, biobehavioral approach.

### Goals (2 to 5 years)

#### Improve bi-directional interaction between neural engineers and clinicians

- Create a dedicated Neuroengineering Session at a major clinical conference.
- Create a Cleveland NEW travel award for students/postdocs to present abstracts at clinical meetings of their choice.
- Develop a mentorship pairing program for neuroengineering students to attend clinical meetings
- Develop a toolkit for undergrads, grad students, post-docs, young investigators, and senior investigators to engage in direct interactions with patients.
- Increase clinician participation in Cleveland NEW:
- Determine which clinicians were invited this year and send email to ask them why they didn’t come, what would entice them to attend.
- Compile list of regional (driving distance or short nonstop flight) clinicians that the group would like to have participating in 2019.
- Describe specific interactions requested from these clinicians.

#### Define views on “augmentation;” a neuroethical framework

- Create a list of potential formal neuroethics training activities and identify one formal neuroethics training activity in which your group will participate and report this intention to Cleveland NEW.

### One degree shifts

- Establish award eligibility for Cleveland NEW travel award and determine target number of awards and funding amount
- Create target list of potential clinical conferences. Request to incorporate more NE in each conference. Request a list of possible mentors for NE grad students
- Compile descriptions of formal BME course-related experiences that already exist.
- Describe mechanisms for:
- inviting patients to course lectures
- inviting students to disease support groups,
- identifying clinical mentors integrating students into neurology/neurosurgery clinics that would allow them to listen to patients’ discussions about their disease and treatments.
- Survey past Cleveland NEW clinicians as to why they didn’t attend. Target clinicians to invite in 2019 who are in the local region. Develop a list of younger clinicians to invite.
- Plan modifications to next Cleveland NEW workshop format to encourage clinician attendance
- Create a list of potential neuroethics training opportunities
- Participate in a neuroethics training

## Theme: Regulatory

### Current state

The regulatory environment in the United States has been changing in a favorable direction since the 2015 workshop. Meeting participants noted recent positive interactions during the pre-submission process, but there remain opportunities to improve communication and to find agreement on the scientific basis for specific regulatory decisions.

#### FDA organization and culture shifts

Dr. Jeffrey Shuren, director of the Center for Devices and Radiological Health at FDA**,** has been working to move the agency towards a “customer service” culture by tracking service performance and making staff accountable. This has encouraged greater attention to improving communication quality and frequency.

The FDA Advancing Regulatory Science Initiative has been building on the achievements of the earlier Critical Path Initiative and other agency programs, and these efforts have changed the way medical products are developed, evaluated and manufactured. This initiative is now expanding its scope to encompass every dimension of regulatory science.

In concert with these organizational and functional changes, interest has been growing at FDA (and CMS) in gaining more knowledge from outside experts such as neuroengineering researchers, clinicians and medical device developers. They are actively seeking clinical, technical and educational information to deepen their understanding and advance their science.

#### Opportunities and obstacles

These trends and regulatory changes open new doors for the neuroengineering community to engage regulatory agencies and establish and maintain two-way communication with their experts. However, discussions during the 2017 workshop sessions indicated that many of the challenges discussed in 2015 are still obstacles today.

For example, Investigational Device Exemption approvals are occurring at a faster rate, but there is still a significant time lapse between IDE and market approval or clearance. Slower approvals cost researcher time and money, and my hinder data collection and ability to meet grant funding milestones.

Improved integration between regulatory and public funding bodies was publicly suggested as one way to streamline the approval process, but such coordination is a challenge due to differing agency regulatory missions. The FDA is looking at safety and efficacy, CMS is looking at patient outcomes, and NIH is focused on innovation.

#### “Reinventing the wheel”

An ongoing challenge for neuroengineers is the IDE submission documentation and review process. Investigators cite a need for a sample or template to provide guidance and standardization for IDE submissions. Such information is not available from FDA. For example, submitters are uncertain about how to classify devices, or whether they can submit a device as a complete system rather than as separate components requiring separate paperwork. Compounding this issue is the lack of consistent two-way communication from regulators to help determine what additional content/testing/action is needed to refine the submission.

### Key factors

#### Lack of an ongoing, established multi-directional communication channel

There are no established mechanisms by which the NE community can provide feedback to regulatory bodies. The lack of two-way conversation hinders full knowledge on both sides.

#### More outbound education and training needed

This would help address knowledge gaps about IDE submissions and regulatory science, which is conducted for a different purpose than research science.

#### Need for the disparate NE community to speak with a unified voice

This would assure that their viewpoint is heard and to effect change. The 2017 workgroup confirmed that this is still a priority and discussed in detail how to develop a consensus opinion on regulatory concerns.

#### Internal knowledge gap among regulators

This can impede the review and approval process. To make the best-informed decisions on new and unique technologies, and to provide useful, science-based feedback, regulatory reviewers should be continually trained in basic science and the most current testing methods.

#### Cost factor

The workgroups feel there is a disassociation at FDA from the direct financial impacts of their process: the work and money invested for each submission, the lost opportunity costs between a three-month good laboratory practice (GLP) and a six-month GLP, or the cost of lost grant-funded research productivity during review period downtime, for example.

### Vision

We envision a seamless integration between funding and regulatory agencies, and two-way communication between these organizations and the NE community, which speaks with one unified voice and is educated in regulatory processes. All stakeholders work together within an expedient, smooth regulatory and reimbursement ecosystem to bring the best neurotechnology products to patients.

### Goals (2 to 5 years)

#### Develop an IDE template or examples to share within the community

- Source the community to collect and collate IDEs.
- Create a NE committee to meet with the FDA and discuss the IDE process.

#### Recommend and identify inter-agency liaisons between federal funding agencies and the FDA to

- Identify partner groups for NE group;
- Provide coaching for submissions; and
- Facilitate parallel submissions.

#### Draft a consensus response to the FDA BCI guidance based on collated comments from the NE community

- Survey NE community for comments on clinical and non-clinical testing

#### Write a formal request that NIH support regulatory science requests for application (RFAs)

- Request NIH allot funds for an expert to guide all funded efforts for the next human studies RFA

### One degree shifts

- Research IDE information to share
- Ask DARPA if funded investigators can share IDE documents publicly
- Make a list of investigators with successful IDE at member institutions
- E-mail members and ask what information if most value to them and adjust content collection accordingly; distribute to Cleveland NEW 2019.
- Develop a VA-FDA liaison, contact DNPMD in CDRH to talk through optimal format of liaison

## Abbreviations

ACA: Affordable Care Act
arXiv: An e-print service in the field of physics, mathematics, computer science, quantitative biology, quantitative finance, statistics, electrical engineering and systems science, and economics
BCI: Brain Computer Interface
bioRxiv: A free online archive and distribution service for unpublished preprints in the life sciences.
BRAIN: Brain Research through Advancing Innovative Neurotechnologies
C3i: Concept to Clinic: Commercializing Innovation
CDRH: Center for Devices and Radiological Health
CMS: Centers from Medicare and Medicaid Services
DBS: Deep Brain Stimulation
DNPMD: Division of Neurological and Physical Medicine Devices
FDA: Food and Drug Administration
FES: Functional Electrical Stimulation
GLP: Good Laboratory Practice
I-CORP: Innovation-Corp
IDE: Investigational Device Exemption
JNE: Journal of Neural Engineering
NANS: North American Neuromodulation Society
NE: Neuroengineering
NEW: Neural Engineering Workshop
NIC: Neural Interfaces Conference
NIH: National Institute of Health
PCOR: Patient-Centered Outcomes Research
PPI: Patient Preference Information
PPP: Public-Private Parternship
PRO: Patient-Reported Outcomes
RFA: Request for Application
VA: Veteran’s Administration
VA: Veteran’s Affairs
